# Timing of Mouse Molar Formation Is Independent of Jaw Length Including Retromolar Space

**DOI:** 10.3390/jdb9010008

**Published:** 2021-03-12

**Authors:** Daisy (Jihyung) Ko, Tess Kelly, Lacey Thompson, Jasmene K. Uppal, Nasim Rostampour, Mark Adam Webb, Ning Zhu, George Belev, Prosanta Mondal, David M. L. Cooper, Julia C. Boughner

**Affiliations:** 1 Department of Anatomy, Physiology & Pharmacology, College of Medicine, University of Saskatchewan, 107 Wiggins Road, Saskatoon, SK S7N 5E5, Canada; daisy.ko@usask.ca (D.K.); tess.kelly@usask.ca (T.K.); lacey.tee1011@gmail.com (L.T.); jku668@mail.usask.ca (J.K.U.); nasimrostampour41@gmail.com (N.R.); davemlcooper@gmail.com (D.M.L.C.); 2 Canadian Light Source, University of Saskatchewan, 44 Innovation Boulevard, Saskatoon, SK S7N 2V3, Canada; Adam.Webb@lightsource.ca (M.A.W.); Ning.Zhu@lightsource.ca (N.Z.); gsb808@mail.usask.ca (G.B.); 3 Clinical Research Support Unit, College of Medicine, University of Saskatchewan, 107 Wiggins Road, Saskatoon, SK S7N 5E5, Canada; prosanta.mondal@usask.ca

**Keywords:** tooth development, molar initiation, craniodental integration, 3D imaging, synchrotron scanning

## Abstract

For humans and other mammals to eat effectively, teeth must develop properly inside the jaw. Deciphering craniodental integration is central to explaining the timely formation of permanent molars, including third molars which are often impacted in humans, and to clarifying how teeth and jaws fit, function and evolve together. A factor long-posited to influence molar onset time is the jaw space available for each molar organ to form within. Here, we tested whether each successive molar initiates only after a minimum threshold of space is created via jaw growth. We used synchrotron-based micro-CT scanning to assess developing molars in situ within jaws of C57BL/6J mice aged E10 to P32, encompassing molar onset to emergence. We compared total jaw, retromolar and molar lengths, and molar onset times, between upper and lower jaws. Initiation time and developmental duration were comparable between molar upper and lower counterparts despite shorter, slower-growing retromolar space in the upper jaw, and despite size differences between upper and lower molars. Timing of molar formation appears unmoved by jaw length including space. Conditions within the dental lamina likely influence molar onset much more than surrounding jaw tissues. We theorize that molar initiation is contingent on sufficient surface area for the physical reorganization of dental epithelium and its invagination of underlying mesenchyme.

## 1. Introduction

The proper development, growth, and evolution of animal bodies require multiple body parts to change together in a coordinated way over time. Explaining the mechanisms underpinning these coordinated changes is foundational to understanding animal evolution and development (evo-devo). A powerful case study of coordination—or “integration”—among body parts is a dentate jaw. Dentitions and jaws appear to have evolved separately from each other, at different times, in prehistoric fossil fishes [1]. Stringent selection pressure for a kinetic jaw outfitted with cutting and grinding tools is surely why the majority of vertebrates living today retain this ancient and revolutionary phenotype [2]. The jaw skeleton and the dentition are both impressively adaptable to a broad range of diets and feeding strategies [3]. Teeth and the jaws that house them also show substantial developmental lability: they may develop, and evolve, in very different ways and in isolation of each other [3,4,5]. Grappling with this paradox of evo-devo synchrony and sovereignty is vital to clarify the mechanisms that generate animal diversity of the face and teeth.

Explaining the developmental mechanisms that coordinate tooth and jaw development is also necessary to improving human dental and oral health. Impaction of third molars, or “wisdom teeth”, is widespread in populations worldwide, much like other common concerns such as dental crowding and malocclusion, and often entails costly, painful interventions including major oral surgery (reviewed in [6,7]). These clinical problems are symptomatic of misfits between dentitions and jawbones, and have little chance of being prevented until we can explain how dental tissues properly fit within jaw tissues as an individual animal develops and grows.

A major obstacle to progress is a technical problem: accurately and precisely seeing, measuring and analyzing in three dimensions (3D) microscopically tiny and radio-translucent tooth organs as they develop in situ within tiny jaw primordia. This 3D work was not feasible until the advent of high-resolution micro-computed tomography (µCT) scanning systems [8] and the ingenious use of tissue contrast agents [9,10]. Indispensable early work in tooth embryology characterized classic stages of tooth organogenesis and morphogenesis (reviewed in [11]). Studies are now visualizing the earliest inception, differentiation, and migration of the cells that will birth the dental lamina [12]. Most of this definitive work has been carried out in mice, the classic biomedical animal model for studying human development and disease. Despite its derived dentition [13,14], the mouse is an apt model of primate including human craniodental developmental processes because these processes are, in general, deeply conserved amongst mammals and across vertebrate classes [3]. 

Traditional laboratory methods to study mouse dentitions can analyze data in only two dimensions (2D), using hand-sectioned, slide-mounted tissues to see tooth organ histology [8,15,16,17]. Because these techniques are labour intensive, time consuming and inherently destructive, they preclude studying structures beyond the tooth organ—namely, surrounding jaw tissues are excluded. For similar pragmatic reasons, studies have historically focused on the lower dentition, limiting data collection from upper teeth. The reason for this focus is the relative ease of experimentally manipulating the mandibular prominence/dentary bone(s). Because of the multi-step convention of cutting, dehydrating and thus potentially distorting already tiny, unmineralized tooth organs, the accuracy and precision of any measurements taken from these tissues are uncertain at best. Additionally, if sliced obliquely to the midline, a 2D section plane may distort and misrepresent a 3D structure. Despite these caveats, more recent studies have used software tools to meticulously reconstruct 2D virtual tissue sections and characterize in 3D developing mouse molars within mineralizing alveolar bone from embryonic day (E)13 to postnatal day (P)26 [18,19,20,21,22,23,24,25]. Additionally, recent in vitro studies have examined molar morphogenesis in 3D using tissue culture. This work has revealed new insights about dental lamina and successive molar development relative to alveolar bone formation, and about bone deposition and resorption at the tooth–bone interface (i.e., at the molar crypt wall) [11,26].

Here we build on this valuable legacy of work by studying tooth morphogenesis in situ within the developing upper and lower jaws. We use direct 3D observations and measurements taken from synchrotron-based µCT scans of a suite of silver-stained [27] C57BL/6J wild-type mouse embryos. The ages of these neonates and pups range from E10 to P32, a period capturing the onset of first (M1), second (M2) and third (M3) molars. We measure the change in molar and jaw mesiodistal lengths relative to one another to investigate the long-standing idea that space created at the back of the growing jaw directly controls when each successive molar begins to form [11,19,20,28,29]. The concept that factors external to a molar organ regulate its onset time contrasts with the model [30] that cues intrinsic to molar mesenchyme underlying the epithelium-derived dental lamina regulate the initiation times of the M2 and M3 (although this model does not account for the mechanism that triggers M1 onset). 

We test the hypothesis that a minimum threshold of retromolar space is required for a molar to initiate. We compare the developmental timing, length, and structure of the upper versus lower dental lamina, molar organs, and retromolar space to glean insights into the evo-devo mechanisms that coordinate changes in the skeletons and dentitions of the mandible and midface. For simplicity, we use “lower jaw” to refer to the mandibular prominence (MdP) and dentary bone/mandible, and “upper jaw” to refer to the maxillary prominence (MxP) and maxilla+premaxilla complex. We often refer to developmental events as occurring “by” a given stage/age to recognize that our scans of molar morphology do not capture data for preceding molecular events. Our quantitative study rebuts the classic hypothesis that space created via jaw growth paces molar initiation and development. Instead of jaw growth, the growth and reorganization of dental epithelium, occurring on a tiny scale, appears more vital to molar onset time. Our work also supports that information inherent to the dental lamina and nascent molar tissues regulates when a given molar initiates.

## 2. Materials and Methods

We collected specimens from our colony of C57BL/6J inbred wild-type mice (stock #000664, Jackson Laboratories). Mice were managed under ethics permit #20110008 in compliance with Canadian Council on Animal Care and University of Saskatchewan regulations. The morning that a vaginal plug was first visible was considered E0.5, which we used to calculate dates of sacrifice and embryo collection. Embryonic stage was determined by tail somite number as well as by limb and craniofacial morphologies as per conventional Theiler staging [31]. Postnatal specimens were collected at ages based on day of birth (P0). We collected specimens aged E10 to P32 (n ≤ 3 specimens/stage, Table 1). As detailed in Raj et al. [27], specimens were 4% paraformaldehyde-fixed, then dehydrated through graded ethanol washes. One day before scanning, specimens were stained overnight with a 1% solution of Protargol (Polysciences; Sigma) tissue contrast agent. Prior to staining, the heads of mice aged P3 and older were cut in half along the sagittal plane to improve stain penetration through skin and bone.

At the Canadian Light Source synchrotron (Saskatoon, SK, Canada), on the BioMedical Imaging and Therapy 05B1-1 beamline, we µCT scanned specimens at 8.75–8.9 µm voxel size using the following parameters: filtered white beam, 4.0 mm aluminum filter, 0.5 mm tin filter, Hamamatsu C9300 camera combined with beam monitor Hamamatsu AA-60. For each scan, a dark field was used to correct for electronic noise, in addition to being used with a flat field to correct for inhomogeneous beam intensity over time.

HCImage software (Hamamatsu) was used to normalize each experimental scan to the dark and flat fields. These normalized scan files were imported into NRecon software (Bruker MicroCT). Here images were reconstructed and optimized via standard protocols to adjust clarity, contrast, and saturation in the region of interest (ROI). Adjustments included misalignment compensation and ring artefact reduction. Next, the digital-format µCT scan files were imported into AMIRA software (Thermo Fisher Scientific, Waltham, MA, USA) to generate 3D volumes, visualize craniodental developmental morphology and anatomy, and collect measurements. In preparation to measure specimens, 3D rendered volumes were aligned as follows. For embryos aged E10-E12, the sagittal plane of view was aligned to the sagittal midline using the gross structures of the head as reference for symmetry; the coronal plane of view was aligned to the straightest and most superior part of neural tube/developing vertebral column. The transverse plane was aligned perpendicular to the sagittal and coronal planes. Due to the lack of homologous landmarks between youngest embryos and older specimens, we used a modified orientation protocol for mice aged E14-P32. For these older specimens, the sagittal plane of view was aligned to the sagittal midline using the gross structures of the head as reference for external symmetry and the palatine process as reference for internal symmetry. The coronal plane of view was aligned to capture upper and lower M1s in the same field of view. The transverse plane of view was aligned to be as parallel to the maxilla as possible while maintaining optimal alignment of sagittal and coronal slices. For all stages/ages, anterior-most and posterior-most points were determined by cross-referencing among coronal, sagittal, and sometimes transverse slices through each specimen.

**Figure 1:**
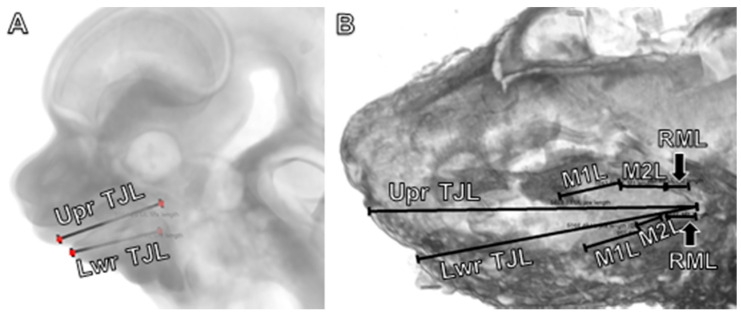
Linear measurements used to define jaw and molar lengths in (**A**) embryonic (E12) and (**B**) perinatal (E18) mice for total jaw length in the upper and lower jaws. Retromolar length is not yet visible in the embryo (**A**). Abbreviations: Lwr, lower jaw; M1L, first molar length, M2L, second molar length, M3L, third molar length; RML, retromolar length; TJL, total jaw length; Upr, upper jaw.

**Figure 2:**
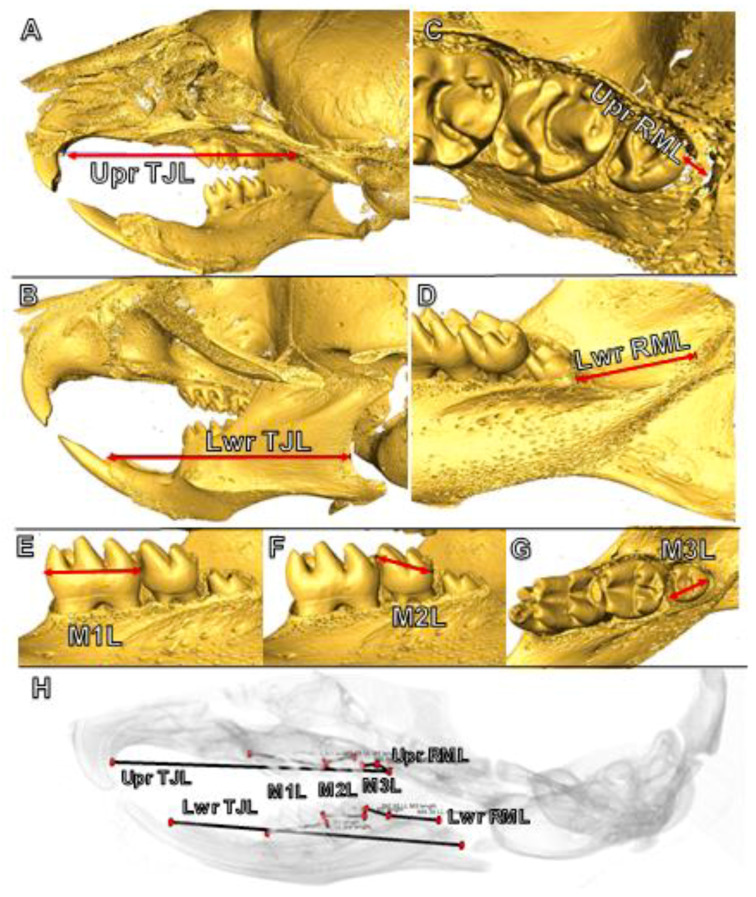
Linear measurements used to define jaw and molar lengths in postnatal mice for total jaw length in the upper (**A**) and lower (**B**) jaws, retromolar length in the maxilla (**C**) and dentary bone (**D**), and first (**E**), second (**F**) and third (**G**) molar lengths. (**H**) A summary of all jaw and molar measurements. Abbreviations: Lwr, lower jaw; M1L, first molar length, M2L, second molar length, M3L, third molar length; RML, retromolar length; TJL, total jaw length; Upr, upper jaw.

In Amira, using sagittal, coronal and transverse virtual planes of view, we measured in 3D upper and lower molar crypt and crown mesiodistal lengths, and total jaw and retromolar space lengths (Figure 1 and Figure 2, Table S1 and Table S2, Video S1) for each quadrant of the mouth. We also assessed stages of molar development. Jaw length including retromolar lengths was measured from anterior to posterior extremes along the same transverse and sagittal planes to avoid artificially inflating the measurement length. Measurement data were analyzed in Excel (Microsoft, Redmond, WA, USA) and SAS (SAS Institute, Cary, NC, USA). We focused our measurements on the left side after confirming via pilot analyses of several pre- and postnatal specimens that left and right side metrics were consistent. For each stage/age, jaw type (i.e., maxillary/upper, mandibular/lower), and measurement type, we averaged measurements taken from left- and right-side structures of each mouse specimen. We used these mean measurements to calculate strengths and directions of correlations among total jaw length, retromolar space and molar length and day of onset. Due to the small sample size of one to three specimens per stage, we analyzed the data with descriptive statistical analyses and graphics. These included plotting for each quadrant (e.g., upper left) the averaged values of independent and dependent variables, and running linear regression models to calculate slope estimates and corresponding standard errors (alpha set to 0.05, two-sided). We also calculated strengths and directions of correlations between retromolar space and molar onset. We included only ratio or proportion data for our single E13 specimen which was truly and inexplicably large, and thus we omitted these raw values from much of our calculation. 

## 3. Results

We report that initiation time was comparable between upper and lower molars. The duration of development was similar among M1, M2 and M3. We observed this outcome even despite size differences among molars, and despite limited retromolar space in the upper jaw that grew more slowly compared to the lower jaw. Our results do not support the hypothesis that jaw length and retromolar space dictate the initiation time of molar development in the mouse. We explain these findings in detail as follows.

### 3.1. Linear Rates of Jaw and Retromolar Growth Contrast with Non-Linear Molar Onset Times

**Figure 3:**
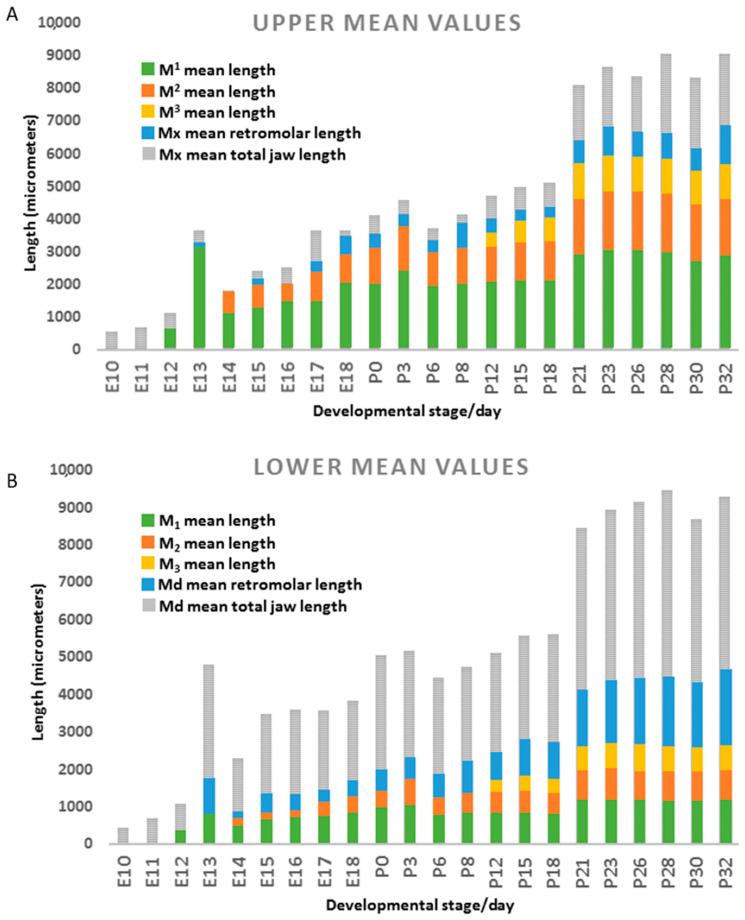
For the (**A**) maxillary prominence (embryos E10–E18) or upper jaw (postnatal mice, P0–P32), and the (**B**) mandibular prominence (E10–E18) and lower jaw (P0–P32), this histogram shows mean mesiodistal lengths of the first (M1L, green), second (M2L, orange) and third (M3L, gold) molar, retromolar space (RML, blue) and entire jaw (TJL, grey). Measurements are averaged among the specimens available for each stage/age. In both jaw types: at E10–E11, no molars have started to form; M1 appears by E12, M2 by E14, and M3 by P12; jaw growth is rapid until E18, after which growth slows, spikes at P21, and slows again (P23–P32). The main differences in the upper jaw are: (1) a much shorter retromolar length (~2–10% of total jaw length) compared to the lower jaw (~10–28% of total jaw length), and (2) upper molars are longer than lower molars. In both jaw types, molars follow a trend of M1 > M2 > M3, but lower molar proportions are closer to M1 > M2 >/= M3. In the upper jaw, the M2 initiates despite very little (~100 µm) of retromolar space.

Although our dataset encompasses root formation and eruption, we focus on molar onset (i.e., the earliest radiographically visible thickening of the dental epithelium) and crown mineralization (Table 2 and Table 3). For each molar type (e.g., M1), onset age is synchronous between upper and lower jaws, and between left and right sides (where left/right data were available) (Figure 3). M1 initiates by E12 based on evidence of epithelial thickening and bud stage tissues, then attains cap stage by E14, early bell stage near E16, and crown mineralization onset by E17. M2 initiates by E14 based on evidence of epithelial thickening, then achieves bud stage by E16, cap stage by E17, and starts mineralizing by E18. For M3, at P3 the crypts of M3 are not yet visible, but at P6 empty-looking crypts are visible as tiny structures in the upper jaw only; and at P8 crypts are visible as larger empty-looking spaces in both jaws. Crown mineralization of M3 starts after P8 and by P12. The M1 crown is complete about 2–4 days (P6–P8) before the M2 crown (P8–P12): the crown mineralization period for each molar is about 2 weeks. For the M3, its crown is complete near P21, closer to 2.5 weeks after initiation (by P4–P6) and about 2 weeks after M3 crown mineralization begins (P8<). Root onset follows shortly after crown completion (Table 2). M1, M2 and M3 are all fully emerged into occlusion around the time that the roots are fully formed with apices closing. By P26 all molars are in occlusion with complete root formation.

Assuming birth around E20 [32], the duration of molar organ morphogenesis and crown mineralization is about 17–19 days for all three molar types in upper and lower jaws. The periods of M1 and M2 formation almost entirely overlap with each other, and overlap with M3 onset and morphogenesis, but barely overlap with the period of M3 crown mineralization (Table 3). Compared to M1 and M2, later M3 emergence is consistent and proportionate to later M3 onset. The duration of crown morphogenesis and mineralization is constant among M1, M2 and M3 and consistent between homologous upper and lower molars. However, the absolute sizes and the relative sizes of mesiodistal crown lengths differ between upper and lower molars (Table 4 and Table 5). Mean absolute lengths of upper M1 and upper M2 generally exceed those of lower M1 and lower M2, respectively (i.e., M^1^ > M_1_ in 15 of 20 stages/ages, and M^2^ > M_2_ in 12 of 18 stages/ages) (Table 4). Conversely, lower M3 length always exceeds that of the upper M3 (i.e., M_3_ > M^3^ in 9/9 stages/ages) (Table 4). In the upper jaw, among fully formed molar crowns (P18 and older) we see smaller ratios of M^2^ and M^3^ lengths relative to M^1^ length (Table 5). These M^2^:M^1^ and M^3^:M^1^ ratios average 61% (range of 60–64%) and 37.3% (36–39%), respectively (Table 5). In contrast, in the lower jaw, M_2_ and M_3_ are on average 68.3% (65–72%) and 58.5% (57–62%) larger than M_1_, respectively (Table 5). Consistently smaller M3:M1 ratios in the upper jaw reflect both an absolutely shorter upper M3 crown and an absolutely longer upper M1 crown. Our findings of jaw-specific differences in M1, M2 and M3 sizes despite comparable durations of development indicate that mesiodistal molar crown length is not solely incumbent on the molar crown formation period. 

### 3.2. Molar Initiation Proceeds Regardless of Jaw Length, Growth Rate, and Retromolar Space Creation

At least one molar must be initiated and present before *bona fide* retromolar (RM) length is present. As such, our study of RM length focuses on ages after M1 onset (E12 onwards), at M2 onset (E14), near M3 onset (P3), and at early M3 mineralization (P12) (Figure 3, Table 4). In the upper jaw, at E14 there is no visible RM length. At P3, upper RM length is 27.8% of M^2^ length and 5% of upper total jaw (TJ) length. At P12, upper RM length is about 94% of M^3^ length and 5.5% of upper TJ length. Generally, from P0 to P32, upper RM length ranges from about 4% to 11% of TJ length, with a mean of almost 6% (Table S3). In the lower jaw, at E14, RM length is about 90% of M_2_ length and almost 10% of TJ length. At P3, lower RM length is almost 84% of M_2_ length and 14% of TJ length. At P12, lower RM length is about 250% of M_3_ length and 19% of TJ length. From P0 to P32, lower RM length ranges from about 14% to 28% of TJ length, with a mean of almost 22% (Table S4). When it could be measured, RM space is consistently greatest in the lower jaw across all seventeen stages/ages (Table 4). Similarly, TJ length is greatest in the lower jaw, with only three exceptions: E10, E12 and E17 (Table 4). In sum, M_2_ initiates with at least 200 µm of RM space distal to M_1_, while M^2^ initiates either with 0 µm of RM space distal to the dental lamina tail or with up to 121 µm of RM space. Overall, compared to the upper jaw, the lower jaw has about 3 to 6 times more retromolar space for M_2_ and M_3_ at their respective ages of initiation.

In both upper and lower jaws, TJ and RM lengths steadily increase from E10 to P3 without spurts of growth before, during, or after the onset time of M1, M2 or M3. Over time, jaw elongation generally shows steepest slopes (fastest growth) from E10 to P3 (Table S5), and gentler slopes (slower growth) from P6 to P32 (Table S6). However, between P18 and P21, in both jaws TJ length increases by about 3000 µm, reaching close to its final maximum length at P32 (Table 4, Figure 3). At all stages, rates of growth for upper and lower TJ lengths are similar (E10-P3: upper left (UL) slope=380 [standard error (SE) = 37], lower left (LL) slope = 441 [SE = 42]; P6-P32: UL = 240 [SE = 31], LL = 221.5 [SE = 27]), with faster rates from E10 to P3 (and possibly up to P5 (Table S3 and Table S4)). 

Conversely, in each jaw type, RM length increases at rates that generally remain constant before and after birth (E10-P3: UL = 11 [SE = 19], LL = 57 [SE = 18]; P6-P32: UL = 14 [SE = 5], LL = 69 [SE = 8]). Compared to TJ lengths, RM lengths show greater variation, with much higher standard error and more low to non-significant p-values (Tables S5 and S6). Additionally unlike TJ lengths, RM length growth rates are slow in the upper jaw (UL = 11 [SE = 20] to 14 [SE = 5]) and noticeably faster in the lower jaw (LL = 57 [SE = 18] to 69 [SE = 8] (Tables S5 and S6)). These slower rates of upper RM growth were seen despite that the upper M1 and M2 are absolutely larger than their lower counterparts, as reported above and elsewhere [25]. 

Lastly, plots of molar length over time for each molar type (e.g., M1/M2) show similar patterns of growth. These patterns are typified by faster increases in size from molar onset to about 7 days later (slope values for left side averages are as follows: E10-P3: M^1^ = 106 [SE = 9.6], M_1_ = 93 [SE = 10], M^2^ = 56 [SE = 15], M_2_ = 76 [SE = 12], (no M3 data before P3); P6-P32: M^1^ = 24 [SE = 5.3], M_1_ = 24 [SE = 4], M^2^ = 22 [SE = 3], M_2_ = 16 [SE = 3]), M^3^ = 18 [SE = 5], M_3_ = 25 [SE = 5.6]) (Table S7 and Table S8).

### 3.3. Total Jaw, Retromolar, and Molar Lengths Are Strongly, Positively Correlated with Each Other

Here we report for upper and lower jaws the Pearson Correlation Coefficients (r) among TJ length, RM length, and M1, M2 and M3 lengths (Table 6). For TJ length, correlations with the other four types of metrics are very strong (r = 0.82 to 0.99) and statistically significant (*p* < 0.01 to *p* < 0.0001). Correlation between TJ length and RM length was weaker in the upper jaw (r = 0.82, 0.83) versus the lower jaw (r = 0.97, 0.99), indicating weaker integration of these two variables in the upper jaw. 

In the lower jaw, M1, M2 and M3 lengths correlated strongly (r = 0.90 to 0.99) with TJ length, indicating that lower jaw length accurately accounts for lower molar length. Among the three lower molars, correlation with TJ length was weakest with M2 (Right (R) M2 r = 0.90, Left (L) M2 r = 0.91) and strongest with M3 (RM3 r = 0.99, LM3 r = 0.98). The strengths of these correlations can be summarized as M2 < M1 < M3. Among upper molars, correlation with TJ length was similar (RM1 r = 0.96, RM2 r = 0.96, RM3 r = 0.998; LM1 r = 0.95, LM2 r = 0.97, LM3 r = 0.95) (*p* ≤ 0.0001). The strengths of these correlations can be summarized as M2 ≈ M1 ≈ M3. 

Next, we explored correlations between RM length and molar lengths (Table 7). In neither jaw did one particular molar type correlate more strongly with RM length. In the lower jaw, correlation strengths were strong (r = 0.79 to 0.97) and, in general, statistically significant (*p* = 0.06 to *p* < 0.0001). Conversely, in the upper jaw, correlation was weaker (0.66 to 0.79; *p* = 0.1 to *p* < 0.001). This outcome indicates weaker integration of upper molars with retromolar space, akin to the results for TJ length showing its weaker correlation with upper molars compared to lower molars. 

### 3.4. Dental Lamina Structure Is Comparable in Upper/Lower Jaws, and Shows Constant Contact with Surface Epithelium

**Figure 4:**
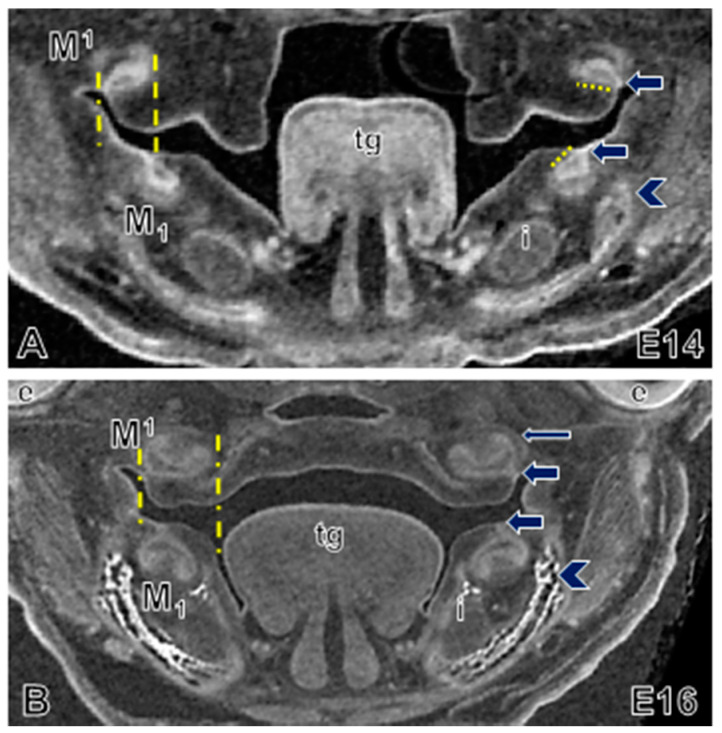
Molar morphogenesis in embryonic wild-type mouse. Coronal view of embryos aged (**A**) E14 and (**B**) E16 showing the lingual tilting (short yellow dotted line, right) of upper and lower M1 organs, and the buccal location of the upper M1 organs at E14. The M1 dental stalks (yellow dashed lines, left, and arrows, right) of the upper molar organs are laterally positioned relative to lower molar organs. By E16 the upper molar organs are wider than the lower organs (yellow dashed lines, left). Mineralizing dentary bone (bright white, arrowhead) is located buccal to the molar and the incisor (i). Long thin arrow (**B**) points to the wall of the dental follicle (crypt). Abbreviations: e, eye; i, incisor; t, tongue.

**Figure 5:**
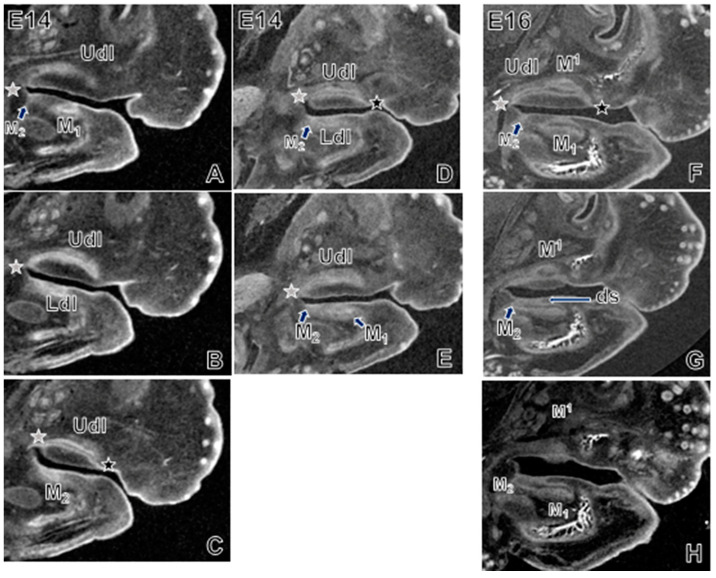
Sagittal plane of embryos aged E14 (**A**–**E**) and E16 (**F**–**H**). In the same plane of section, the upper and lower M1 organs and dental lamina are at different depths. Versus the lower jaw that aligns with the Current Model [11] of dental lamina structure, the appearance of the dental lamina in the upper jaw conforms to the Classical Model [11] (black star: contact of anterior end of upper dental lamina with surface epithelium; grey star, contact of posterior end of dental epithelium with surface epithelium). However, this conformity is actually an artefact of the more steeply bucco-lingual inclination of the upper molar organ. The onset of M2 is just visible as a small epithelial invagination in the lower jaw (arrows/labels, panels **A**,**D**,**F**), which even at E14 and clearly by E16 is visible an extension of the M1 organ and dental epithelium (arrows, **G**,**H**). Abbreviations: ds, dental stalk; Ldl, lower dental lamina; M, molar; Udl, upper dental lamina.

Lastly, we explored the structure and orientation of molar organs and the dental lamina from before M1 onset (E10) and onwards. At E14 and E16 (Figure 4, Video S2), the upper and lower M1s were tilted lingually (as seen in the coronal plane of section) with the dental stalk located buccal to the rest of the M1 organ. Additionally at E14, M^1^ was located buccal to M_1_ (Figure 4A). The different appearance of the upper versus lower dental lamina in the same sagittal section (Figure 5A–C, Video S2) reflects in part that the upper lamina is buccal to the lower lamina (Video S2). By E16, the upper and lower M1 and M2 organs were positioned directly atop each other (Figure 4B). A lingual incline of M^1^ and M^2^ persisted at E17 and E18, but was not seen in the lower molar counterparts, which were positioned along a straight axis (in coronal plane). Although we also saw this arrangement of upper molars positioned lateral (or buccal) to lower counterparts (also reported by Peterka et al. [25]), buccolateral position varied according to the depth of the coronal plane at which the embryo/molars were viewed.

In coronal view, the molar lamina resembles a spherical structure of peripheral and inner epithelium surrounded by condensed mesenchyme on its aboral aspect (Figure 4, Video S2). At E14 and E16, the dental stalk, which is derived from an epithelial invagination of mesenchyme, is clearly visible as a continuous structure that anchors the M1 and M2 organs to the adjacent dental epithelium along the mesiodistal length of the dental lamina (Figure 4, thick arrows; Figure 5G,H, “ds”). Each molar forms on either side of its invagination site. This site is located at about the midpoint along each molar organ’s length. In the sagittal view, only along the midline of the mass of the dental lamina was it in continuous contact with the overlying surface epithelium (Figure 5G,H). Further buccally or lingually, only the dental stalks of M1 and M2 connect with the surface epithelium. At the time of M2 onset, a virtual section through the midline of the M1 shows that the enamel organ, which is located near the surface epithelium buds, off the tail end of the M1 (Figure 5G,H). This budding is accompanied by an epithelial invagination at the position of M2 (Figure 5A,C,D,F).

Depending on the angle of the virtual section in the sagittal plane, the lamina has two appearances. In the same plane of view, the lower lamina looks like an elongate structure that runs parallel to and maintains contact with the surface epithelium (Figure 5G,H). Conversely, the upper lamina looks like an arched structure that contacts the surface epithelium only at its mesial and distal ends (upper jaw) (Figure 5C,D,F). For the more lingually-tilted (as well as more buccally located) upper lamina, the section plane is at an angle to the perfect midsagittal plane (Video S3). When reoriented in a perfect cranial–caudal axis along the sagittal plane, the upper dental lamina looks comparable to its lower counterpart. We contend that the arched structure is an artefact of the plane of section. Lastly, in a transverse plane of section, the posterior tail of the lower dental lamina merges with the lingual oral epithelium of the mandible. In this same plane, the posterior tail of the upper dental lamina merges with the epithelium along the buccal aspect of each palatal shelf (Video S4). At E16 the lamina between M1 and M2 was clearly continuous. As early as E17–E18, we detected a bony septum starting to form between M1 and M2, similar to reports by an earlier study [21] of this septum at birth (P0).

## 4. Discussion

### 4.1. Enabling Molar Initiation Does Not Appear to Be the Main Impetus for Jaw Growth and Space Creation

In our sample of mouse specimens aged E10 to P32, the initiation times and durations of M1, M2 and M3 development were consistent regardless of differences in retromolar (RM) space between upper and lower jaws. For both the upper and lower jaws, growth rates were faster up until shortly after birth, around P3. After P3, jaw growth rates were slower. We did not see bursts of jaw growth around the time of the earliest visible molar thickening or crypt [20]. Retromolar space was available about one week prior to M3 onset, negating the idea that the pause between M2 and M3 onset is to allow sufficient jaw growth [19]. Faster jaw growth during embryonic stages may reflect rapid change related not to molar development but instead to factors including secondary palate formation (E13–E16) [25]. Comparable rates of upper and lower total jaw (TJ) growth contrasted with staccato molar onset times. Successive molars initiated with progressively longer pauses between them, despite that the M3 was the shortest molar. Collectively, these findings undermine the idea that space creation through jaw growth triggers molar initiation. 

The strong and significant correlations that we saw between TJ length and RM length, as well as molar lengths, indicate strong integration of these structures during morphogenesis and growth. However, RM length was consistently the weakest and most variable correlate with TJ and molar lengths, particularly in the upper jaw, which had the least RM space. These findings suggest that RM length is a minor influence on developing molars. The retromolar length was also not reliably predicted by stage/age (Table S3 and S4), despite that molar length was accurately estimated by developmental day. In the face of equivalent upper and lower molar onset times, weaker correlations of RM length with molar and TJ lengths underscore that retromolar space does not dictate when molar development begins.

The primary impetus for (postnatal) jaw growth does not appear to be enabling molar initiation. Rather, this impetus appears to be to maintain the symmetry of the mandible and midfacial skeleton, and ensure proper occlusion of the upper and lower dentitions [33]. Counter to past reports of faster lower jaw growth, specifically of the lower diastema, from E14 to E18 [25] we did not see faster growth of the lower jaw compared to its upper counterpart; although in the lower jaw, retromolar length grew about five times faster. We suspect that these different results between studies are due to different measurement protocols designed to target distinct research aims. 

### 4.2. Molar Crown Length Does Not Appear to Influence Molar Development Duration

Our observations that M^1^ was generally longer than M_1_, and M^2^ was longer than M_2_, while M^3^ was always shorter than M_3_, are consistent with earlier work [25]. Our results for the onset times and durations of molar development align well with past reports based on other inbred wild-type laboratory mouse strains, e.g., ICR/Jcl [32] and CD1 [19,21] (reviewed in [19]). Differences among our collective findings likely reflect technical variation in data collection methodologies, as well as biological variation within and among genetic backgrounds and breeding colonies [34]. Our results also agree with past work [19] reporting that different molar types (e.g., M1, M3) develop through homologous stages at comparable tempos and durations. To paraphrase Chlastakova and colleagues [19], we report a constant period of mouse molar crown formation despite that the M3 is considerably smaller than the M1 and, to which we would add, despite size differences between upper and lower molars.

### 4.3. Molar Initiation Is Preceded by Minimal Dental Lamina Growth

Our results support past work [35] indicating that in the mouse mandibular molar, the tooth organ grows into the surrounding mesenchyme, i.e., away from the oral cavity, and maintains contact with surface epithelium via the dental stalk. Chlastakova et al. [19] describe that M3 arises from folding on the lingual side of the M2/dental lamina. Unfortunately, we could not see the M3 extension clearly enough to comment. However, our scans at E14 and E16 show M2 as a distinct invagination of dental epithelium that, towards its midline, is connected to the mass of the M1 organ and dental lamina. This structure of the initiating M2 is akin to what we saw for M1 (i.e., a direct outgrowth of the dental lamina with clear epithelial invagination into underlying mesenchyme). 

Our results also concur with recent work defining the structure and process by which new molars arise from the distal part of the outer epithelium of the preceding molar. This origin includes a dual contribution of superficial dental epithelium and molar tail epithelium from the preceding molar organ [11]. Gaete and colleagues [11] proposed this structure as a new “Current Model” of the shape and progression of the dental lamina, and as an alternative to the “Classical Model” of successional molar formation. The Classical Model defines the dental lamina as diving beneath the oral epithelium, with mesenchyme separating the lamina from epithelium, while the lamina grows parallel to the surface. The Current Model describes the dental lamina as a structure that runs beneath but in continuous contact with the oral epithelium. Our own 3D scan datasets (Videos S2 and S3, Figure 5) generally conform to the Current Model. Along the lateral margins of the dental lamina, we saw a gap between the lamina and oral epithelium; but along the lamina’s midline, the dental stalk tethered the epithelium of each molar organ to the surface epithelium. The dental lamina appeared to extend backward at the same time as the invagination of the dental epithelium at the location of the next initiating molar. This observation emphasizes that molar initiation is not contingent on much if any retromolar space. 

### 4.4. A Role for the Propagation of Dental Epithelium in the Timing of Molar Onset: A Hypothesis 

Based on our findings, we suggest that molar onset is not constrained by jaw length *sensu stricto* but instead by oral epithelium length. Epithelial folding happens across areas as brief as 10 to 100 micrometers, possibly with inputs from condensing mesenchyme [36,37,38]. We propose that while molar onset is impartial to the jaw and retromolar length, the tail of the dental lamina must be able to extend at least marginally in order to birth a successive molar. In this regard, we suggest that the propagation of oral and subsequently dental epithelium is most vital to molar initiation. We hypothesize that only marginal elongation of the jaw primordium and, later, the jawbone, is important for molar initiation. We propose that this marginal growth allows the oral/dental epithelium and dental lamina to achieve a minimum length required for the ribbon of tissue to buckle, catalyzing invagination of the epithelium into the underlying mesenchyme [39]. 

This hypothesis is supported by recent biophysical modeling of molar organogenesis, particularly epithelial folding. The lingual tilting of the upper and lower molar organs that we observed was also reported by Takigawa-Imamura et al. [36]. The tilt of the enamel epithelium supports a biomechanical model [36] of asymmetrical epithelial dynamics at early molar morphogenesis (bud stage) (E14), and suggests that this tilt is less important at later stages (cap stage) (E16). In this biomechanical modeling of mouse molar odontogenesis, during bud-to-cap transition, this tilting levels out somewhat [36]. In our dataset, at E16 the upper M2 organs were not only tilted lingually but also positioned more lingually than were the lower M2 organs (Video S2). At the same time, bone was mineralizing inferolingually and inferobucally to the lower M1, and inferolingually (and slightly inferobucally) to the upper M1. We wonder if this lingual tilt of the tooth organ also helps facilitate the local alveolar bone formation, where shearing forces created by mesenchymal tissues growing at varying speeds trigger cell differentiation and subsequent intramembranous ossification [20,40]. The idea that dental tissue intrinsically regulates molar initiation is supported by findings that the dental follicle produces its own adjacent alveolar bone [41,42,43] and then instructs this bone to remodel at the tooth–bone interface (TBI) during molar histogenesis and eruption [20,23,44]. Although the activities of osteoclasts and osteoblasts at the TBI appear to influence molar organ growth [26], at the time of initiation, molar primordia are free from surrounding bone. Only at the bell stage does bone gradually begin to encapsulate each molar within a bony socket ([19] and reviewed in [21]). These studies bolster our conjecture that initiation time manifests via the progression and organization of the molar lamina, not via the progression of jawbone growth and space creation. 

### 4.5. Implications for Human Dental Development and Dental Health

Increasingly longer latency between successively initiating molars, particularly M2 and M3, is seen in mice as well as humans and other primates. Some African apes and monkeys present with extended pauses of months or years between the initiation of adjacent molars while also showing constant jaw growth rates [45,46]. In these primates, space also does not appear to constrain molar initiation [47]. Additionally seen in primates including humans and in rodents, initiation times are equivalent between upper and lower molars even while the maxilla shows much less retromolar space than the dentary bone. We also observed in mice that spurts of jaw growth occur only after the onset of M1, M2 and M3, near the emergence times of M1 and M2. Together, these results indicate that in at least some mammalian groups, jaw space and length do not trigger molar initiation. Rather, these findings emphasize the distinction that jaw length and space are vital for proper molar eruption and emergence into occlusion, but do not seem essential for timely molar initiation and morphogenesis. Applying our findings to human wisdom teeth: earlier M3 development increases the likelihood of proper M3 eruption [48], but the size of total jaw length and retromolar space may not explain earlier or later M3 development. To explain the etiology of other misfits between human dentitions and jawbones, such as dental crowding and malocclusion, our findings imply that it is more informative to study variables acting during late stages of tooth development (i.e., eruption) that help properly integrate teeth within the jawbone and the dental arcade. 

### 4.6. Notes on Future Studies of Dental Lamina and Molar Developmental Morphologies

Based on our findings, we offer some recommendations for future studies of tooth development. First, our dataset shows that the structure of the dental lamina can look very different depending on how a virtual section is angled relative to the sagittal plane. Thus how exactly the dental lamina is viewed is integral to defining its structure and explaining its development. Depth of section is also important in terms of defining the positions of upper and lower molar organs relative to each other. In our experience with 3D scan data of E14 specimens, the buccolingual laterality of the M1 organ is more or less exaggerated depending on the anteroposterior section in the coronal plane of view (Video S2): as the dental lamina lengthens (e.g., E14–E17) it gently angles buccolaterally, as alluded to by others during E13.5–E15 [25]. This variation in buccolingual positions of upper and lower molar primordia may help explain different observations among studies.

Next, desktop µCT [49] in complement with tissue contrast agents (e.g., [50]) is increasing the accessibility and speed with which developmental morphology can be imaged and studied in 3D. As such, the jaw and tooth measurements that we defined here using synchrotron µCT are a potential foundation and template for future quantitative studies of craniodental morphogenesis. Limitations of our technique include difficulty distinguishing soft from hard tissue in older specimens aged E18 and later, and thus addressing questions about dental lamina extension and the organization of M2 and M3 outgrowths. Other study limitations are small sample sizes per stage/age group, and potential bias due to measurement definitions. 

Lastly, for sound reasons including ease of access, past studies have favored the mandibular jaw primordium and thus the lower molar row for experimental work including in vitro organ cultures (e.g., [11,30]). Our work suggests that aside from jaw-specific variation in molar lengths and proportions, the basic structure of the dental lamina is conserved between upper and lower molar rows. Thus, the mandibular prominence remains a good proxy to model molar development in general. However, because mouse/permanent molars are additional teeth [13], our findings may differ from those based on permanent teeth that replace deciduous precursors. Despite its synapomorphies, the mouse model system remains invaluable for understanding fundamental deeply conserved processes in mammalian early tooth morphogenesis. An ongoing issue with using mice to model mammalian craniodental development and growth is that the mouse jawbone is so under-mineralized compared to humans and other primates, in which permanent molars initiate and form within fully mineralized jawbones. Studying diphyodont animal models such as wallaby [50] is an excellent complementary approach to address this question.

## 5. Conclusions

Despite jaw-specific differences in crown size and retromolar growth, the age and duration of initiation, morphogenesis and mineralization are comparable between upper and lower molars. Proportions and correlation strengths of retromolar space with molar length differ between upper and lower jaws, and among M1, M2 and M3, likely reflecting morphological and ontogenetic variation among these bones and teeth. Thus molar initiation time does not appear to be modulated by jaw growth and subsequent retromolar space creation. Our results support that conditions within the dental lamina exert greater influence on molar initiation than surrounding external jaw tissues, including space. We propose that triggers of successive molar initiation are instead biomechanical and developmental–genetic cues intrinsic to the dental lamina, and contingent on the progression and physical restructuring of the dental epithelium to enable its invagination into underlying mesenchyme.

## Figures and Tables

**Table 1:** Sample sizes of the embryonic (E) and postnatal (P) mouse specimens studied here, where the number (e.g., 10) is either the day post-conception (E) or post-birth (P).

Prenatal Mice		Postnatal Mice			
Stage	# Specimens	Age	# Specimens	Age	# Specimens
E10	3	P0	1	P26	3
E11	1	P3	3	P28	1
E12	3	P6	3	P30	2
E13	1	P8	2	P32	3
E14	2	P12	3		
E15	3	P15	2		
E16	1	P18	2		
E17	3	P21	3		
E18	3	P23	2		

**Table 2:** Stages of earliest radiographically visible tooth organ onset, crown completion, root onset and completion, and tooth emergence for the first (M1), second (M2) and third (M3) molars of both the upper and lower jaws. Abbreviations: –, to/between; <, earlier than; >, later than; ≤, earlier or at.

Molar	Organ Onset	Crown Complete	Root Onset	Root Complete	Emergence
**M1**	E12	<P6–P8	P8	P21–P23	>P18–P21
**M2**	E14	>P8–P12	>P8, <P15	P23–P26	>P18–P21
**M3**	≤P4–P6	P21	>P21–P23	P28–P30	P23–P26

**Table 3:** Bar chart of prenatal and postnatal stages of onset (leftmost end of each row). This chart indicates the start (*) and completion (^) of crown mineralization, and emergence (e) of first (M1), second (M2) and third molar (M3) teeth in C57BL/6J mice. Staging is based on our image data from embryonic (E) stage E10 to postnatal (P) age 26. Total duration of development is comparable among all three molars (M1, M2, M3). Periods of M1 and M2 morphogenesis and crown formation largely overlap with each other but do not overlap with those of M3 because of its later onset by about 10 days after birth. * = start of crown mineralization, ^ = crown completion, e = emergence into occlusion.

E12	E14	E16	E18	P0	P2	P4	P6	P8	P10	P12-	-P18	P20	P22	P24	P26
**M1**		* E17					^ P6–8				eP18–21				
	**M2**		*					^ P8–12				e/P21			
						**M3**		* P8<				^ P21		eP23–26	

**Table 4:** Upper (M^x^/Upr) and lower (M_x_/Lwr) first (M1), second (M2) and third (M3) molar, total jaw and retromolar mean mesiodistal lengths in micrometers (µm) for each prenatal (E) and postnatal (P) stage studied here. Per stage, all measurements for left and right sides were averaged. Typically, M^1^ > M_1_ (15/20 stages) and M^2^ > M_2_ (12/18 stages), while M_3_ > M^3^ for 9/9 stages (*italicized values* = Upr > Lwr). Total jaw length was longer in the lower jaw with three exceptions (*italicized*) at E10, E12 and E17. Retromolar space was always longer in the lower jaw. “-” indicates that at least one molar was not yet forming and no value could be measured for that stage/age group. * The inexplicably large absolute metrics for E13 are excluded from all analyses, only some data for E13 ratios and proportions are included.

	M1 Length µm		M2 Length µm		M3 Length µm		Total Jaw µm		Retromolar µm	
Stage	Upr	Lwr	Upr	Lwr	Upr	Lwr	Upr	Lwr	Upr	Lwr
**E10**	-	-	-	-	-	-	*560.8*	450.5	-	-
**E11**	-	-	-	-	-	-	685.2	702.7	-	-
**E12**	362.3	424.6	-	-	-	-	*1117.4*	1070.8	-	-
E13 *	*1886.1*	999.6	-	-	-	-	3646.2	4799.4	85.2	1193.8
**E14**	*657.1*	588.3	*388.5*	250.0	-	-	1809.1	2304.3	-	226.0
**E15**	750.1	808.3	*431.8*	223.9	-	-	2436.7	3493.5	120.8	651.2
**E16**	865.8	883.6	*322.8*	226.3	-	-	2519.1	3612.3	-	527.5
**E17**	861.6	917.9	*564.1*	484.9	-	-	*3643.9*	3576.9	183.5	408.3
**E18**	*1219.8*	1027.0	512.6	539.7	-	-	3663.6	3827.6	351.6	545.8
**P0**	1195.4	1229.2	*662.0*	524.7	-	-	4109.7	5057.1	258.0	739.7
**P3**	*1437.4*	1300.0	828.9	864.6	-	-	4590.2	5171.8	230.3	724.9
**P6**	*1160.5*	938.8	617.0	620.3	-	-	3715.1	4431.4	216.1	790.3
**P8**	*1188.7*	1009.4	679.0	696.2	-	-	4158.4	4721.3	451.7	1075.0
**P12**	*1231.1*	1018.5	641.7	710.3	276.6	385.9	4712.0	5102.3	259.0	966.3
**P15**	*1246.8*	1033.2	723.8	739.1	402.6	485.7	4992.5	5570.4	199.0	1258.3
**P18**	*1247.3*	1002.0	*744.9*	685.4	431.1	492.4	5109.3	5603.9	215.1	1241.5
**P21**	*1734.1*	1457.4	*1046.1*	993.3	658.6	827.5	8102.8	8447.0	416.4	1880.8
**P23**	*1816.3*	1471.5	*1104.4*	1054.1	654.4	847.4	8646.6	8955.3	553.3	2137.3
**P26**	*1819.2*	1478.1	*1089.9*	957.0	658.7	911.5	8355.2	9138.0	449.1	2220.5
**P28**	*1781.9*	1423.2	*1085.2*	1015.7	660.4	822.2	9071.2	9472.0	481.6	2370.5
**P30**	*1622.8*	1446.9	*1036.8*	975.8	631.1	824.8	8328.7	8688.8	426.3	2198.7
**P32**	*1720.6*	1462.4	*1048.3*	980.0	657.3	865.5	9045.5	9292.9	721.9	2579.3

**Table 5:** Ratios of averaged first (M1), second (M2) and third (M3) molar mesiodistal lengths from stages/ages E14 to P32, for left and right sides of each jaw type (upper; lower). The closer a value is to 100, the greater the parity of M2 or M3 length relative to M1. Ratios tend to increase with stage because, relative to M1 (which is crown complete by P8), the M2 and M3 elongate until about P12 and P21, respectively. We saw a trend of smaller M2:M1 ratios in the lower jaw until about birth (P0), after which M2:M1 ratio was smaller in the upper jaw. Conversely, M3:M1 ratios were consistently smaller in the upper jaw. “-” = a molar was not yet forming and could not be measured thus the ratio is absent for that stage/age group.

	Upper Jaw		Lower Jaw	
Stage	M^2^:M^1^	M^3^:M^1^	M_2_:M_1_	M_3_:M_1_
**E14**	59	-	43	-
**E15**	58	-	28	-
**E16**	37	-	26	-
**E17**	66	-	53	-
**E18**	42	-	53	-
**P0**	55	-	43	-
**P3**	58	-	67	-
**P6**	53	-	66	-
**P8**	57	-	69	-
**P12**	52	23	70	38
**P15**	58	32	72	47
**P18**	60	35	68	49
**P21**	60	38	68	57
**P23**	61	36	72	58
**P26**	60	36	65	62
**P28**	61	37	71	58
**P30**	64	39	67	57
**P32**	61	38	67	59

**Table 6:** Pearson’s Correlation Coefficient (r) of total jaw length (TJL) with retromolar length (RML), and molar lengths (M1, M2, M3), showing strong and significant correlations in all four quadrants among upper and lower jaws. Strength of correlation between TJL and RML was highest in the mandible (0.97–0.99) versus the maxilla (0.82–0.83).

**Upper Left TJL vs.**	**Upper Right TJL vs.**
RML 0.83, *p* < 0.0001, n = 16	RML 0.82, *p* < 0.01, n = 9
M^1^ 0.95, *p* < 0.0001, n = 19	M^1^ 0.96, *p* < 0.0001, n = 12
M^2^ 0.97, *p* < 0.0001, n = 18	M^2^ 0.96, *p* < 0.0001, n = 11
M^3^ 0.95, *p* = 0.0001, n = 9	M^3^ 0.998, *p* < 0.0001, n = 5
**Lower Left TJL vs.**	**Lower Right TJL vs.**
RML 0.99, *p* < 0.0001, n = 18	RML 0.97, *p* < 0.0001, n = 11
M_1_ 0.94, *p* < 0.0001, n = 19	M_1_ 0.96, *p* < 0.0001, n = 12
M_2_ 0.91, *p* < 0.0001, n = 18	M_2_ 0.90, *p* = 0.0002, n = 11
M_3_ 0.98, *p* < 0.001, n = 9	M_3_ 0.99, *p* = 0.001, n = 5

**Table 7:** Pearson’s Correlation Coefficient (r) of retromolar length (RML) with molar lengths (M1, M2, M3), showing strong and significant correlations (*) for most but not all molars all four quadrants among upper and lower jaws. In all four quadrants, significance of correlation was lowest for M3 with RML. There is no clear pattern where one molar type correlates most strongly with RML.

**Upper Left RML vs.**	**Upper Right RML vs.**
M^1^ 0.79, *p* < 0.001 *, n = 16	M^1^ 0.76, *p* = 0.02, n = 9
M^2^ 0.78, *p* < 0.001 *, n = 16	M^2^ 0.66, *p* = 0.05, n = 9
M^3^ 0.75, *p* = 0.02, n = 9	M^3^ 0.78, *p* = 0.1, n = 5
**Lower Left RML vs.**	**Lower Right RML vs.**
M_1_ 0.87, *p* < 0.0001 *, n = 18	M_1_ 0.85, *p* < 0.001 *, n = 11
M_2_ 0.89, *p* < 0.0001 *, n = 18	M_2_ 0.79, *p* < 0.01 *, n = 11
M_3_ 0.97, *p* < 0.001 *, n = 9	M_3_ 0.85, *p* = 0.06, n = 5

## Data Availability

Data are contained within the article or supplementary material. Scan data will be made available on FaceBase (https://www.facebase.org/, accessed on 5 March 2021) and is available at DOI: 10.25550/1-Y732.

## Supplementary Materials

The following are available online at https://www.mdpi.com/2221-3759/9/1/8/s1, Table S1: Raw measurement data for mouse molar, total jaw, and retromolar lengths, Table S2: Descriptions of measurements used for embryonic and perinatal/postnatal specimens ranging from embryonic day (E) 10 to postnatal day (P) 32, Table S3: From E14 to P32, total upper jaw length in micrometres, and percent of total upper jaw length occupied by upper molar mesiodistal crypt or crown lengths, as well as retromolar jaw length and the diastema and incisor region, Table S4: From E14 to P32, total lower jaw length in micrometres, and percent of total lower jaw length occupied by lower molar mesiodistal crypt or crown lengths, as well as retromolar jaw length and the diastema and incisor region, Table S5: From E10 to P3, linear regression model showing that stage/age accurately predicts molar and jaw lengths but not retromolar lengths before and shortly after birth, Table S6: From P6 to P32, linear regression model showing that stage/age accurately predicts all measurements with the exception of upper retromolar length, Table S7: Linear regression model showing that jaw length accurately predicts molar length, Table S8: Linear regression model showing that, in general, retromolar length accurately predicts molar length, particularly in the mandible. Video S1: Synchrotron micro-CT scan of E16 mouse showing linear measurement, Video S2: Synchrotron micro-CT scan of E16 mouse, sagittal and coronal views, Video S3: Synchrotron micro-CT scan of E16 mouse, upper dental lamina in situ, Video S4: Synchrotron micro-CT scan of E16 mouse, transverse view.
