# Correction: Ko et al. Timing of Mouse Molar Formation Is Independent of Jaw Length Including Retromolar Space. *J. Dev. Biol.* 2021, *9*, 8

**DOI:** 10.3390/jdb13010007

**Published:** 2025-02-28

**Authors:** Daisy (Jihyung) Ko, Tess Kelly, Lacey Thompson, Jasmene K. Uppal, Nasim Rostampour, Mark Adam Webb, Ning Zhu, George Belev, Prosanta Mondal, David M. L. Cooper, Julia C. Boughner

**Affiliations:** 1 Department of Anatomy, Physiology & Pharmacology, College of Medicine, University of Saskatchewan, 107 Wiggins Road, Saskatoon, SK S7N 5E5, Canada; daisy.ko@usask.ca (D.K.); tess.kelly@usask.ca (T.K.); lacey.tee1011@gmail.com (L.T.); jku668@mail.usask.ca (J.K.U.); nasimrostampour41@gmail.com (N.R.); davemlcooper@gmail.com (D.M.L.C.); 2 Canadian Light Source, University of Saskatchewan, 44 Innovation Boulevard, Saskatoon, SK S7N 2V3, Canada; Adam.Webb@lightsource.ca (M.A.W.); Ning.Zhu@lightsource.ca (N.Z.); gsb808@mail.usask.ca (G.B.); 3 Clinical Research Support Unit, College of Medicine, University of Saskatchewan, 107 Wiggins Road, Saskatoon, SK S7N 5E5, Canada; prosanta.mondal@usask.ca

There was an error in the original publication [1]. Table S1 was an incomplete version, and Videos S1–S4, along with Tables S2–S8, were mistakenly omitted from the Supplementary Materials. The corrected Tables and Videos appear below:

Supplementary Video S1:

https://drive.google.com/file/d/1pzx9nbtbivb3NK3HV6ZPray897EFwgly/view

Supplementary Video S2:

https://drive.google.com/file/d/16t1nWSlpS0rRw3BajXGPcnIxIWl6vKIX/view

Supplementary Video S3:

https://drive.google.com/file/d/1S1EAxmTMn1WS0CLGb_8Ygi3UiXoEMEAZ/view

Supplementary Video S4:

https://drive.google.com/file/d/1Rn5wRTbKHYNddbn3-HVJ8bsdl4mxBF0A/view

Table S1. Raw measurement data for mouse molar, total jaw, and retromolar lengths. (https://drive.google.com/file/d/1pFWmqRkFrHfzMSoS_5wRE-cuTsoUUAiQ/view), and Tables S2–S8 appear below.

A correction has been made to the Supplementary Materials, and they are available online at https://www.mdpi.com/2221-3759/9/1/8/s1. The authors state that the scientific conclusions are unaffected. This correction was approved by the Academic Editor. The original publication has also been updated.

**Table S2:** Descriptions of measurements used for embryonic and perinatal/postnatal specimens ranging from embryonic day (E) 10 to postnatal day (P) 32.

Measurement Ages	Measurement Parameters	Notes
E10-18: Lower jaw length (Figure 4) P0-P32: Lower jaw length (Figure 5)	Anterior-most midline point on (mineralized tissue^1^ of) the mandible, measured to the; Anterior-most point of subcondylar incisive Most anterior-superior midline point of the alveolar rim between the incisors, to the; Anterior-most point of subcondylar incisive	^1.^ E10-12 measure mandibular prominences (no mineralized jaw tissue was present yet). ^2.^ The absolute anteriorposterior value was taken, without any rostral-caudal and medial-lateral angulation, to avoid artificially inflating length. i.e., antero-posterior maximums were take along the same sagittal and transverse planes. ^3.^ E10-12 measure maxillary prominences (mineralized jaw tissue was not present yet). ^4.^ At E10 no molars have visibly initiated. ^5.^ Retromolar length is by nature contingent on the presence of at least a first molar organ. ^6.^ E14-16 use the most anterior point of the curve of the posterior aspect of the ramus as a proxy, because the jaw is not sufficiently mineralized to see a mandibular foramen.
E10-18: Upper jaw length (Figure 4) P0-P32: Upper jaw length (Figure 5)	Anterior-most point of the maxilla^3^, measured to the; Posterior-most point of the maxilla^2^. Most anterior-inferior point of the premaxilla between the incisors, to the; Posterior-most point of the maxilla^2^.	
E10-P32: Molar length (Figure 5)	Anterior-most point of molar organ or mineralizing/ed crown, measured to the; Posterior-most point of the same molar organ or mineralizing/ed crown^4^.	
E12-P32: Retromolar length^5^ (Figures 4 and 5)	Posterior landmark of latest-forming molar organ/crown, measured to the; Posterior-most point of the mandibular foramen^2,6^ of the mandible.	

**Table S3:** From stages/ages E14 to P32, total upper jaw length in micrometres (µm), and the percent (%) of total upper jaw length occupied by upper first (M1), second (M2) and third (M3) molar mesiodistal crypt or crown lengths, as well as retromolar jaw length (3–35%) and remaining (i.e., non-molar, non retromolar) jaw length (~40–55%) for the diastema and incisor region. For every stage/age: there is space distal to the last-initiated molar; no notable increase in retromolar (RM) jaw length prior to M3 onset, and; M1 > M2 > M3 although M1 is longer and M3 shorter compared to their lower counterparts) Measurements are from the upper left side only, averaged for each stage/age (see Table 5 for full data used in this table).

Stage/Age	Upper TJL (µm)	MD Molar Length as % of Upper TJL			% of Upper TJL	
		M1	M2	M3	RML	Remaining
**E12**	1117.42	32.4	-	-	-	-
E13 *	3646.20	51.7	-	-	2.34	46.0
**E14**	1809.08	36.3	21.5	-	-	42.2
**E15**	2436.74	30.8	17.7	-	4.96	46.5
**E16**	2519.10	34.4	12.8	-	-	52.8
**E17**	3643.86	24.0	15.9	-	5.03	55.1
**E18**	3663.62	33.3	14.0	-	9.60	43.1
**P0**	4109.65	29.1	16.1	-	6.28	48.5
**P3**	4590.23	31.3	18.0	-	5.02	45.7
**P6**	3715.06	31.2	16.6	-	5.82	46.4
**P8**	4158.42	28.6	16.3	-	10.86	44.2
**P12**	4712.02	26.1	13.6	5.9	5.50	48.9
**P15**	4992.52	25.0	14.5	8.1	3.98	48.4
**P18**	5109.28	24.4	14.6	8.4	4.21	48.4
**P21**	8102.80	21.4	13.0	8.1	5.14	52.4
**P23**	8646.60	21.0	12.8	7.6	6.40	52.2
**P26**	8355.17	21.8	13.1	7.9	5.38	51.8
**P28**	9071.21	19.6	12.0	7.3	5.31	55.8
**P30**	8328.67	19.5	12.5	7.6	5.12	55.3
**P32**	9045.49	19.0	11.6	7.3	7.98	54.1

“-” indicates that at least one molar was not yet forming and thus no value could be measured for that stage/age group. * As noted in the main text and tables, E13 is inexplicably large and we use these data for proportions only. Abbreviations: MD, mesiodistal; RML, retromolar length; TJL, total jaw length.

**Table S4:** From stages/ages E14 to P32, total lower jaw length in micrometres (µm), and the percent (%) of total lower jaw length occupied by lower first (M1), second (M2) and third (M3) molar mesiodistal crypt or crown lengths, as well as retromolar jaw length (~15–30%) and remaining (i.e., non-molar, non retromolar) jaw length (~35–50%) for the diastema and incisor region. For every stage/age: there is space distal to the last-initiated molar (i.e., no shortage of space); no notable increase in retromolar jaw length before M3 onset (P12); also, the entire molar row length is ~30–40% of the total lower jaw length, and M1 > M2 > M3. Measurements are from the lower left side only, averaged for each stage/age (see Table 5 for full data used in this table).

Stage/Age	Lower TJL (µm)	MD Molar Length as % of Lower TJL			% of Lower TJL	
		M1	M2	M3	RML	Remaining
**E12**	1070.77	39.65	-	-		
E13 *	4799.40	20.8	-	-		
**E14**	2304.25	25.5	10.9	-	9.81	53.79
**E15**	3493.51	23.1	6.4	-	18.64	51.86
**E16**	3612.25	24.5	6.3	-	14.60	54.60
**E17**	3576.85	25.9	13.7	-	11.41	48.99
**E18**	3827.62	26.8	14.1	-	14.24	44.86
**P0**	5057.14	24.3	10.4	-	14.63	50.67
**P3**	5171.79	25.1	16.7	-	14.02	44.18
**P6**	4431.37	21.2	14.0	-	17.83	53.03
**P8**	4721.25	21.4	14.8	-	22.77	41.03
**P12**	5102.26	20.0	14.0	7.6	18.94	39.46
**P15**	5570.41	18.5	13.3	8.7	22.59	36.91
**P18**	5603.85	18.0	12.2	8.8	22.15	38.85
**P21**	8447.00	17.3	11.8	9.8	22.27	38.83
**P23**	8955.31	16.4	11.8	9.5	23.87	38.43
**P26**	9137.99	16.2	10.5	10.0	24.3	39.00
**P28**	9472.03	15.0	10.7	8.7	25.03	40.57
**P30**	8688.78	16.6	11.2	9.5	25.31	37.40
**P32**	9292.92	15.7	10.5	9.3	27.76	36.75

“-” indicates that at least one molar was not yet forming and could not be measured. * As noted in the main text and tables, E13 is inexplicably large and we use these data for proportions only. Abbreviations: MD, mesiodistal; RML, retromolar length; TJL, total jaw length.

**Table S5:** From E10 to P3, linear regression model for day (independent variable) versus molar length (M1, M2, M3) showing that stage/age accurately predicts molar and jaw lengths but not retromolar lengths before and shortly after birth. M3 is not included as no data for this variable exists at these young stages/ages.

Dependent Variable	Jaw Type	Estimate (SE)	*p*-Value
M1	Md	92.77 (9.92)	<0.0001
	Mx	106.03 (9.64)	<0.0001
M2	Md	76.04 (11.70)	0.0013
	Mx	55.80 (14.79)	0.0130
Total jaw length	Md	440.88 (42.04)	<0.0001
	Mx	379.70 (37.06)	<0.0001
Retromolar length	Md	56.73 (17.77)	0.0242 *
	Mx	10.91 (19.60)	0.6167 *

All quadrants are left side. *, not significant; Md, mandible; Mx, maxilla; SE, standard error.

**Table S6:** From P6 to P32, linear regression model for day (independent variable) versus molar length (M1, M2, M3), total jaw length, and retromolar length, showing that stage/age accurately predicts all measurements with the exception of upper retromolar length.

Dependent Variable	Jaw Type	Estimate (SE)	*p*-Value
M1	Md	23.90 (4.17)	0.0003
	Mx	23.89 (5.25)	0.0004
M2	Md	16.02 (2.83)	0.0003
	Mx	21.86 (3.23)	<0.0001
M3	Md	25.01 (5.63)	0.0030
	Mx	17.65 (4.65)	0.0055
Total jaw length	Md	221.54 (26.71)	<0.0001
	Mx	240.02 (31.03)	<0.0001
Retromolar length	Md	69.03 (7.49)	<0.0001
	Mx	14.31 (4.97)	0.0182 *

All quadrants are left side. *, not significant; Md, mandible; Mx, maxilla; SE, standard error.

**Table S7:** Linear regression model for total jaw length (independent variable) versus molar length (M1, M2, M3) showing that jaw length accurately predicts molar length.

Dependent Variable	Jaw Type	Estimate (SE)	*p*-Value
M1	Md	0.11 (0.01)	<0.0001
	Mx	0.15 (0.01)	0.0001
M2	Md	0.11 (0.01)	<0.0001
	Mx	0.10 (0.01)	<0.0001
M3	Md	0.11 (0.01)	<0.0001
	Mx	0.07 (0.01)	0.0001

All quadrants are left side. Md, mandible; Mx, maxilla; SE, standard error.

**Table S8:** Linear regression model for retromolar length (independent variable) versus molar length (M1, M2, M3) showing that, in general, retromolar length accurately predicts molar length, particularly in the mandible.

Dependent Variable	Jaw Type	Estimate (SE)	*p*-Value
M1	Md	0.31 (0.04)	<0.0001
	Mx	1.53 (0.32)	0.0003
M2	Md	0.33 (0.04)	<0.0001
	Mx	1.07 (0.23)	0.0003
M3	Md	0.34 (0.03)	<0.0001
	Mx	0.57 (0.19)	0.02 *

All quadrants are left side. *, not significant; Md, mandible; Mx, maxilla; SE, standard error.
