# Supplementary material for: Timing of Mouse Molar Formation Is Independent of Jaw Length Including Retromolar Space
Source: J Dev Biol. 2021 Mar 12;9(1):8. doi: 10.3390/jdb9010008 (PMC8006249; doi:10.3390/jdb9010008)
Supplement: Supplementary file 1 [file jdb-09-00008-s001.pdf]

**Table S1. Raw measurement data for mouse molar, jaw and retromolar length**

**Each row represents a different specimen**

**Legend:**

*E=embryonic day* *0=zero (could be measured; was equal to 0)* *9999=absent/could not be measured*

*P=postnatal day* *LL=lower left* *M1=1st molar* *M2=2nd molar* *M3=3rd molar* *RM=retromolar* *LR=lower right*

| Age | Excel "day" | LL jaw length | M1 LL length | M2 LL length | M3 LL length | LL RM length | LR jaw length | M1 LR length | M2 LR length | M3 LR length | LR RM length |
|---|---|---|---|---|---|---|---|---|---|---|---|
| E10 | -9 | 554.07 | 9999 | 9999 | 9999 | 9999 | 526.35 | 9999 | 9999 | 9999 | 9999 |
| E10 | -9 | 333.16 | 9999 | 9999 | 9999 | 9999 | 383.28 | 9999 | 9999 | 9999 | 9999 |
| E10 | -9 | 462.41 | 9999 | 9999 | 9999 | 9999 | 443.56 | 9999 | 9999 | 9999 | 9999 |
| E11 | -8 | 694.93 | 9999 | 9999 | 9999 | 9999 | 710.39 | 9999 | 9999 | 9999 | 9999 |
| E12 | -7 | 1063.96 | 9999 | 9999 | 9999 | 9999 | 1017.5 | 9999 | 9999 | 9999 | 9999 |
| E12 | -7 | 1190.58 | 418.94 | 9999 | 9999 | 9999 | 1014.62 | 430.25 | 9999 | 9999 | 9999 |
| E12 | -7 | 1096.2 | 9999 | 9999 | 9999 | 9999 | 1041.78 | 9999 | 9999 | 9999 | 9999 |
| E13 | -6 | 4827.15 | 1068.38 | 9999 | 9999 | 1181.68 | 4771.66 | 930.81 | 9999 | 9999 | 1205.82 |
| E14 | -5 | 2424.53 | 568.59 | 256.84 | 9999 | 240.35 | 2359.11 | 656.81 | 217.93 | 9999 | 148.28 |
| E14 | -5 | 2185.00 | 618.63 | 240.34 | 9999 | 271.24 | 2248.38 | 508.99 | 285.03 | 9999 | 244.19 |
| E15 | -4 | 9999 | 816.18 | 179.88 | 9999 | 9999 | 3499.76 | 799.03 | 250.78 | 9999 | 661.5 |
| E15 | -4 | 3471.62 | 780.83 | 290.7 | 9999 | 636.23 | 3531.02 | 789.79 | 307.87 | 9999 | 670.75 |
| E15 | -4 | 9999 | 808.2 | 151.6 | 9999 | 9999 | 9999 | 855.72 | 162.51 | 9999 | 9999 |
| E16 | -3 | 3621.35 | 934.4 | 214.97 | 9999 | 526.31 | 3603.15 | 832.82 | 237.61 | 9999 | 528.59 |
| E17 | -2 | 3328.38 | 925.65 | 584.26 | 9999 | 147.47 | 9999 | 939.38 | 555.38 | 9999 | 9999 |
| E17 | -2 | 9999 | 9999 | 9999 | 9999 | 9999 | 3231.03 | 890.37 | 400 | 9999 | 332.69 |
| E17 | -2 | 4516.94 | 916.27 | 400.13 | 9999 | 820.22 | 9999 | 9999 | 9999 | 9999 | 9999 |
| E18 | -1 | 3855.78 | 1101.54 | 472.85 | 9999 | 630.79 | 3867.24 | 1043.61 | 639.54 | 9999 | 537.51 |
| E18 | -1 | 9999 | 9999 | 9999 | 9999 | 9999 | 9999 | 977.09 | 612.76 | 9999 | 281.96 |
| E18 | -1 | 3720.22 | 985.81 | 433.47 | 9999 | 732.81 | 9999 | 9999 | 9999 | 9999 | 9999 |
| P0=birth | 0 | 5057.14 | 1229.23 | 524.67 | 9999 | 739.73 | 9999 | 9999 | 9999 | 9999 | 9999 |
| P3 | 3 | 9999 | 9999 | 9999 | 9999 | 9999 | 5263.26 | 1305.58 | 1010.59 | 9999 | 553.59 |
| P3 | 3 | 5152.41 | 1298.53 | 803.28 | 9999 | 809.97 | 9999 | 9999 | 9999 | 9999 | 9999 |
| P3 | 3 | 9999 | 9999 | 9999 | 9999 | 9999 | 5119.06 | 1297.52 | 841.36 | 9999 | 725.89 |
| P6 | 6 | 9999 | 935.05 | 632.6 | 9999 | 856.7 | 9999 | 9999 | 9999 | 9999 | 9999 |
| P6 | 6 | 4431.37 | 952.84 | 628.81 | 9999 | 723.93 | 9999 | 9999 | 9999 | 9999 | 9999 |
| P6 | 6 | 9999 | 928.42 | 599.36 | 9999 | 9999 | 9999 | 9999 | 9999 | 9999 | 9999 |
| P8 | 8 | 4743.33 | 1030.73 | 730.87 | 9999 | 1131.43 | 9999 | 9999 | 9999 | 9999 | 9999 |
| P8 | 8 | 4699.17 | 988.08 | 661.49 | 9999 | 1018.54 | 9999 | 9999 | 9999 | 9999 | 9999 |
| P12 | 12 | 5121.90 | 1008.81 | 785.95 | 380.55 | 9999 | 9999 | 9999 | 9999 | 9999 | 9999 |
| P12 | 12 | 5064.25 | 1023.21 | 669.69 | 384.17 | 9999 | 9999 | 9999 | 9999 | 9999 | 9999 |
| P12 | 12 | 5120.62 | 1023.39 | 675.33 | 392.90 | 966.3 | 9999 | 9999 | 9999 | 9999 | 9999 |
| P15 | 15 | 5557.89 | 1051.13 | 786.35 | 490.90 | 1300.64 | 9999 | 9999 | 9999 | 9999 | 9999 |
| P15 | 15 | 5582.92 | 1015.20 | 691.90 | 480.52 | 1215.92 | 9999 | 9999 | 9999 | 9999 | 9999 |
| P18 | 18 | 5706.14 | 994.57 | 682.62 | 461.13 | 1247.51 | 9999 | 9999 | 9999 | 9999 | 9999 |

**Table S1. Raw measurement data for mouse molar, jaw and retromolar length**

| | | | | | | | | | | | |
|---|---|---|---|---|---|---|---|---|---|---|---|
| **P18** | 18 | 9999 | 9999 | 9999 | 9999 | 9999 | 5501.56 | 1009.47 | 688.15 | 523.67 | 1235.43 |
| **P21** | 21 | 9999 | 9999 | 9999 | 9999 | 9999 | 8224.09 | 1448.11 | 977.2 | 812.7 | 1853.63 |
| **P21** | 21 | 9999 | 9999 | 9999 | 9999 | 9999 | 8901.26 | 1490.45 | 1057.69 | 921.6 | 1857.27 |
| **P21** | 21 | 8331.33 | 1445.52 | 969.05 | 787.93 | 1906.16 | 9999 | 9999 | 9999 | 9999 | 9999 |
| **P23** | 23 | 9999 | 9999 | 9999 | 9999 | 9999 | 9152.44 | 1477.54 | 1070.28 | 875.79 | 2054.32 |
| **P23** | 23 | 8758.18 | 1465.4 | 1037.94 | 818.95 | 2220.3 | 9999 | 9999 | 9999 | 9999 | 9999 |
| **P26** | 26 | 9415 | 1483.2 | 898.57 | 926.9 | 2324.27 | 9999 | 9999 | 9999 | 9999 | 9999 |
| **P26** | 26 | 9999 | 9999 | 9999 | 9999 | 9999 | 9275.71 | 1476.27 | 1082.85 | 912.57 | 2305.31 |
| **P26** | 26 | 9999 | 9999 | 9999 | 9999 | 9999 | 8446.23 | 1469.71 | 947.91 | 879.53 | 1928.03 |
| **P28** | 28 | 9472.03 | 1423.21 | 1015.65 | 822.22 | 2370.53 | 9999 | 9999 | 9999 | 9999 | 9999 |
| **P30** | 30 | 8923.14 | 1456.43 | 1025.18 | 833.08 | 2146.82 | 9999 | 9999 | 9999 | 9999 | 9999 |
| **P30** | 30 | 8454.41 | 1437.41 | 926.41 | 816.47 | 2250.51 | 9999 | 9999 | 9999 | 9999 | 9999 |
| **P32** | 32 | 9343.29 | 1460.04 | 1014.71 | 820.06 | 2468.67 | 9999 | 9999 | 9999 | 9999 | 9999 |
| **P32** | 32 | 9999 | 9999 | 9999 | 9999 | 9999 | 9999 | 1469.41 | 927.52 | 880.57 | 9999 |
| **P32** | 32 | 9999 | 9999 | 9999 | 9999 | 9999 | 9242.54 | 1460.02 | 962.95 | 941.42 | 2689.82 |

**Table S1. Raw measurement data for mouse molar, jaw and retromolar length**

| | | *UL=upper left* | | | | | *UR=upper right* | | | | |
|---|---|---|---|---|---|---|---|---|---|---|---|
| **Age** | **Excel "day"** | **UL jaw length** | **M1 UL length** | **M2 UL length** | **M3 UL length** | **UL RM length** | **UR jaw length** | **M1 UR length** | **M2 UR length** | **M3 UR length** | **UR RM length** |
| **E10** | -9 | 586.73 | 9999 | 9999 | 9999 | 9999 | 600.28 | 9999 | 9999 | 9999 | 9999 |
| **E10** | -9 | 477.95 | 9999 | 9999 | 9999 | 9999 | 495.68 | 9999 | 9999 | 9999 | 9999 |
| **E10** | -9 | 613.33 | 9999 | 9999 | 9999 | 9999 | 590.87 | 9999 | 9999 | 9999 | 9999 |
| **E11** | -8 | 667.68 | 9999 | 9999 | 9999 | 9999 | 702.66 | 9999 | 9999 | 9999 | 9999 |
| **E12** | -7 | 1005.57 | 9999 | 9999 | 9999 | 9999 | 1036.75 | 9999 | 9999 | 9999 | 9999 |
| **E12** | -7 | 9999 | 420.43 | 9999 | 9999 | 9999 | 1422.45 | 439.76 | 9999 | 9999 | 9999 |
| **E12** | -7 | 1094.2 | 299.84 | 9999 | 9999 | 9999 | 1095.68 | 289.25 | 9999 | 9999 | 9999 |
| **E13** | -6 | 3655.58 | 1904.69 | 9999 | 9999 | 71.07 | 3636.81 | 1867.48 | 9999 | 9999 | 99.35 |
| **E14** | -5 | 1819.92 | 671.91 | 419.43 | 9999 | 9999 | 1816.44 | 643.57 | 399.04 | 9999 | 9999 |
| **E14** | -5 | 1796.68 | 687.30 | 365.98 | 9999 | 9999 | 1803.26 | 625.59 | 369.60 | 9999 | 9999 |
| **E15** | -4 | 2443.88 | 757.85 | 423.44 | 9999 | 9999 | 2466.33 | 741.1 | 428.56 | 9999 | 9999 |
| **E15** | -4 | 2446.24 | 774.53 | 435.56 | 9999 | 112.95 | 2447.88 | 779.47 | 426.92 | 9999 | 128.63 |
| **E15** | -4 | 2430.3 | 696.98 | 440.18 | 9999 | 9999 | 2385.79 | 750.42 | 436.27 | 9999 | 9999 |
| **E16** | -3 | 2504.78 | 858.35 | 249.07 | 9999 | 9999 | 2533.42 | 873.23 | 396.53 | 9999 | 0.00 |
| **E17** | -2 | 3246.30 | 892.36 | 519.18 | 9999 | 170.84 | 3256.22 | 957.86 | 644.54 | 9999 | 9999 |
| **E17** | -2 | 9999 | 9999 | 9999 | 9999 | 9999 | 3206.91 | 778.82 | 613.01 | 9999 | 97.29 |
| **E17** | -2 | 4866.02 | 817.22 | 479.49 | 9999 | 368.37 | 9999 | 9999 | 9999 | 9999 | 9999 |
| **E18** | -1 | 3682.79 | 1330.96 | 506.28 | 9999 | 368.90 | 3674.30 | 1353.80 | 425.34 | 9999 | 443.19 |
| **E18** | -1 | 9999 | 9999 | 9999 | 9999 | 9999 | 9999 | 1150.61 | 610.91 | 9999 | 225.52 |
| **E18** | -1 | 3623.1 | 1043.91 | 9999 | 9999 | 9999 | 9999 | 9999 | 9999 | 9999 | 9999 |
| **P0=birth** | 0 | 4109.65 | 1195.42 | 661.96 | 9999 | 257.98 | 9999 | 9999 | 9999 | 9999 | 9999 |
| **P3** | 3 | 9999 | 9999 | 9999 | 9999 | 9999 | 4805.95 | 1476.28 | 870.3 | 9999 | 312.5 |
| **P3** | 3 | 4470.24 | 1413.99 | 778.51 | 9999 | 222.28 | 9999 | 9999 | 9999 | 9999 | 9999 |
| **P3** | 3 | 9999 | 9999 | 9999 | 9999 | 9999 | 4614.47 | 1445.12 | 888.28 | 9999 | 163.95 |
| **P6** | 6 | 3754.61 | 1156.7 | 649.86 | 9999 | 170.27 | 9999 | 9999 | 9999 | 9999 | 9999 |
| **P6** | 6 | 3949.98 | 1166.90 | 617.01 | 9999 | 274.80 | 9999 | 9999 | 9999 | 9999 | 9999 |
| **P6** | 6 | 3440.59 | 1157.90 | 584.11 | 9999 | 203.14 | 9999 | 9999 | 9999 | 9999 | 9999 |
| **P8** | 8 | 4150.73 | 1207.73 | 737.71 | 9999 | 395.82 | 9999 | 9999 | 9999 | 9999 | 9999 |
| **P8** | 8 | 4166.11 | 1169.75 | 620.30 | 9999 | 507.51 | 9999 | 9999 | 9999 | 9999 | 9999 |
| **P12** | 12 | 4723.36 | 1226.78 | 740.35 | 317.74 | 195.2 | 9999 | 9999 | 9999 | 9999 | 9999 |
| **P12** | 12 | 4801.43 | 1240.64 | 679.22 | 171.74 | 199.30 | 9999 | 9999 | 9999 | 9999 | 9999 |
| **P12** | 12 | 4611.28 | 1225.96 | 505.65 | 340.24 | 382.37 | 9999 | 9999 | 9999 | 9999 | 9999 |
| **P15** | 15 | 5140.60 | 1270.06 | 749.61 | 421.77 | 215.57 | 9999 | 9999 | 9999 | 9999 | 9999 |
| **P15** | 15 | 4844.45 | 1223.53 | 697.98 | 383.49 | 182.27 | 9999 | 9999 | 9999 | 9999 | 9999 |
| **P18** | 18 | 5151.13 | 1246.03 | 738.13 | 414.70 | 234.23 | 9999 | 9999 | 9999 | 9999 | 9999 |

**Table S1. Raw measurement data for mouse molar, jaw and retromolar length**

| | | | | | | | | | | | |
|---|---|---|---|---|---|---|---|---|---|---|---|
| **P18** | 18 | 9999 | 9999 | 9999 | 9999 | 9999 | 5067.43 | 1248.56 | 751.57 | 447.56 | 195.94 |
| **P21** | 21 | 9999 | 9999 | 9999 | 9999 | 9999 | 7746.38 | 1754.16 | 1008.15 | 658.97 | 315.39 |
| **P21** | 21 | 9999 | 9999 | 9999 | 9999 | 9999 | 8033.65 | 1838.8 | 1132.9 | 653.83 | 260.14 |
| **P21** | 21 | 8315.59 | 1671.63 | 1021.6 | 660.77 | 545.02 | 9999 | 9999 | 9999 | 9999 | 9999 |
| **P23** | 23 | 9999 | 9999 | 9999 | 9999 | 9999 | 8256.31 | 1812.08 | 1092.75 | 669.23 | 559.78 |
| **P23** | 23 | 9036.89 | 1820.54 | 1116.06 | 639.61 | 546.74 | 9999 | 9999 | 9999 | 9999 | 9999 |
| **P26** | 26 | 8649.62 | 1853.1 | 1096.72 | 662.15 | 486.71 | 9999 | 9999 | 9999 | 9999 | 9999 |
| **P26** | 26 | 9999 | 9999 | 9999 | 9999 | 9999 | 8256.56 | 1833.96 | 1142.56 | 668.26 | 478.56 |
| **P26** | 26 | 9999 | 9999 | 9999 | 9999 | 9999 | 7864.87 | 1736.7 | 1023.4 | 642.15 | 344.22 |
| **P28** | 28 | 9071.21 | 1781.91 | 1085.15 | 660.4 | 481.63 | 9999 | 9999 | 9999 | 9999 | 9999 |
| **P30** | 30 | 8657.08 | 1663.77 | 1104.29 | 637.97 | 437.1 | 9999 | 9999 | 9999 | 9999 | 9999 |
| **P30** | 30 | 8000.25 | 1581.8 | 969.34 | 624.31 | 415.43 | 9999 | 9999 | 9999 | 9999 | 9999 |
| **P32** | 32 | 9474.34 | 1798.04 | 1109.89 | 612.75 | 799.8 | 9999 | 9999 | 9999 | 9999 | 9999 |
| **P32** | 32 | 9999 | 9999 | 9999 | 9999 | 9999 | 8554.05 | 1631.56 | 969.16 | 708.42 | 9999 |
| **P32** | 32 | 9999 | 9999 | 9999 | 9999 | 9999 | 8679.24 | 1654.77 | 1004.07 | 695.24 | 643.96 |

**Table S1. Raw measurement data for mouse molar, jaw and retromolar length**

**Table S1. Raw measurement data for mouse molar, jaw and retromolar length**
